# P-2097. Genomic basis of Oxacillin- and Cefoxitin-Susceptible PBP2a-Positive *Staphylococcus aureus*

**DOI:** 10.1093/ofid/ofae631.2253

**Published:** 2025-01-29

**Authors:** Lili Tao

**Affiliations:** Vanderbilt University Medical Center, Nashville, Tennessee

## Abstract

**Background:**

Methicillin-resistance *Staphylococcus aureus* (MRSA) is resistant to all ß-lactam antibiotics except for the fifth-generation cephalosporin drug, ceftaroline. The *mecA* gene, which encodes an alternative penicillin-binding protein, PBP2a, is responsible for the high-level resistance to methicillin. However, *mecA* positive, oxacillin- and cefoxitin susceptible MRSA has been reported, with limited understanding of the genomic basis behind this contradictory phenomenon. Here, we studied the molecular base of a “stealth” MRSA from clinical specimen.

**Methods:**

A MRSA isolate was routinely isolated from a deep wound specimen, which was positive for PBP2a by lateral flow assay but negative for cefoxitin screen test and susceptible to oxacillin (MIC of 0.5 ug/ml) by VITEK 2 AST-GP75 card. It was also susceptible to cefoxitin using disk diffusion method (diameter of 26 mm). The “stealth” MRSA isolate was sequenced on illumina NovaSeq 6000 platform using 150 bp paired end sequencing. Bacteria genome was assembled and annotated using pipelines on BV-BRC (https://www.bv-brc.org/).

**Results:**

Genomic sequencing revealed a point mutation (G to A) in the *mecA* gene resulting in a Gly599Ser substitution located in the transpeptidase domain of PBP2a, which is close to the active site of Ser403 on the 3D structure of the protein. Additionally, an insertion in the methicillin resistance regulatory sensor-transducer *mecR1* gene was identified, resulting in a frameshift mutation, and predicted to cause premature termination. However, the sequence of frame shift mutation in *mecR1* has been described and was not associated with reversed cefoxitin susceptibility. Therefore, the point mutation in *mecA* gene is most likely responsible for loss of function of PBP2a, and lead to susceptible cefoxitin and oxacillin results despite a positive PBP2a reaction.

**Conclusion:**

Whole genome sequencing is valuable to understand the genomic basis of unusual phenotypic testing results. The reversion of “stealth” MRSA strain to a high-level oxacillin- and cefoxitin-resistant strain has been reported, which highlights the importance of considering tests for *mecA* or PBP2a in routine laboratory practice to avoid the risk of missing such strains.

**Disclosures:**

All Authors: No reported disclosures

